# Nanofluids for heat transfer: an engineering approach

**DOI:** 10.1186/1556-276X-6-182

**Published:** 2011-02-28

**Authors:** Elena V Timofeeva, Wenhua Yu, David M France, Dileep Singh, Jules L Routbort

**Affiliations:** 1 Energy Systems Division, Argonne National Laboratory, Argonne, IL 60439, USA; 2 Department of Mechanical and Industrial Engineering, University of Illinois at Chicago, Chicago, IL 60607, USA; 3 Nuclear Engineering Division, Argonne National Laboratory, Argonne, IL 60439, USA

## Abstract

An overview of systematic studies that address the complexity of nanofluid systems and advance the understanding of nanoscale contributions to viscosity, thermal conductivity, and cooling efficiency of nanofluids is presented. A nanoparticle suspension is considered as a three-phase system including the solid phase (nanoparticles), the liquid phase (fluid media), and the interfacial phase, which contributes significantly to the system properties because of its extremely high surface-to-volume ratio in nanofluids. The systems engineering approach was applied to nanofluid design resulting in a detailed assessment of various parameters in the multivariable nanofluid systems. The relative importance of nanofluid parameters for heat transfer evaluated in this article allows engineering nanofluids with desired set of properties.

## Introduction

Suspensions of solid submicron- and nanometer-sized particles in various fluids (also called nanofluids) have been considered for applications as advanced heat transfer fluids for almost two decades. However, due to the wide variety and the complexity of the nanofluid systems, no agreement has been achieved on the magnitude of potential benefits of using nanofluids for heat transfer applications. Large volume of studies devoted to characterization of individual thermo-physical properties of nanofluids, such as thermal conductivity, viscosity, and agglomeration of nanoparticles, has been summarized in a number of review articles [1-9].

Evaluation of cooling efficiency, i.e., ability to remove heat from the heat source, includes assessing flow regime-dependent contributions from thermal conductivity, viscosity, specific heat, and density of the fluid and also depends on the applied flow regime. The studies devoted to evaluation of the heat transfer performance of nanofluids are scarce and inconclusive compared to the studies on the thermo-physical properties of various nanofluids indicating a significant gap between fundamental research and practical applications of nanofluids for thermal management.

In this article we present a summary of systematic experimental studies of both thermo-physical properties and heat transfer in nanofluids. We believe that the underestimated complexity and the controversy of nanofluid systems is related to the solid/liquid boundary layers between nanoparticles and the liquid, which at significant surface area of nanoparticles contribute to the fluid properties, resulting in three-phase systems. The approach to nanofluids as three-phase systems (instead of traditional consideration of nanofluids as two-phase systems of solid and liquid) allows for deeper understanding of correlations between the engineering parameters, nanofluid properties, and cooling performance. The factors contributing to the fluid cooling efficiency are discussed first, followed by a review of nanofluid engineering parameters and a brief analysis of their contributions to basic thermo-physical properties. Finally, a systems engineering approach is used to describe how various nanofluid parameters contribute to the cooling performance. The latter also offers insights into the principles of the efficient nanofluid design.

### Cooling efficiency of nanofluids

The initial promise of nanofluids as advanced heat transfer fluids was based on the increased thermal conductivity of nanoparticle suspensions. Low thermal conductivity of conventional fluids improves when the solid particles are added. However, the magnitudes of the effects reported in the literature are scattered from few percent (as predicted by effective medium theory (EMT) [10-12]) to hundred percents per volume concentration of nanoparticles (i.e., abnormal enhancements [4,13,14]). Theoretical works exploring the mechanisms that could be responsible for abnormally enhanced thermal conductivities are widely presented in the literature [2,15].

Unfortunately it is not always realized that the thermal conductivity is not the only property that determines the efficiency of heat transfer in the system. In the forced flow systems the coolant is pumped through the pipes of a heat exchanger, introducing convective heat transfer mechanisms and pumping power penalties. Efficiencies of various liquid coolants depend on the fluid properties and the flow mode (laminar or turbulent) and can be estimated from the fluid dynamics equations [16]. In the case of hydrodynamically and thermally fully developed laminar flow, the heat transfer coefficient (*h*) is proportional to the thermal conductivity (*k*), and independent of the flow velocity (within the acceptable range of inlet/outlet temperature difference) [17]:

(1) h∝k

An alternative merit criterion for laminar flow [18] was suggested, for situation, when the tube diameter can be increased for the nanofluid to result in the same heat transfer coefficient:

(2) $$\frac{\mu_{\text{eff}}}{\mu_{0}}\approx 1+C_{\mu }\phi ;\;\frac{k_{\text{eff}}}{k_{0}}\approx 1+C_{k}\phi ;\;C_{\mu }/C_{k}<4$$

where *φ *is the particle volume concentration, *μ *is the dynamic viscosity of the nanofluid (eff) and the base fluid (0), and *C*_*μ *_and *C*_*k *_are viscosity and thermal conductivity enhancement coefficients, determined from experimental viscosity and thermal conductivity ratios. However, it is not very practical when efficiencies of two fluids are compared in the same system geometry (i.e., tube diameter).

In turbulent flow regime the heat transfer rate (based on the Dittus-Boelter equation for heating applications) is dependent not only upon the thermal conductivity (*k*), but also on the density (*ρ*), specific heat (*c*_*p*_), viscosity (*μ*), and flow velocity (*V*) [16]:

(3) $$h\propto \rho^{4/5}c_{p}^{2/5}\mu^{-2/5}k^{3/5}V^{4/5}$$

Introduction of nanoparticles to the fluid affects all of thermo-physical properties and should be accounted for in the nanofluid evaluations [18,19]. Density and specific heat are proportional to the volume ratio of solid and liquid in the system, generally with density increasing and specific heat decreasing with addition of nanoparticles to the fluid. According to Equation (3) the increase in density, specific heat, and thermal conductivity of nanofluids favors the heat transfer coefficient; however, the well-described increase in the viscosity of nanoparticle suspensions is not beneficial for heat transfer. The velocity term in Equation (3) also represents the pumping power penalties resulting from the increased viscosity of nanofluids [16,20].

For comparing two liquid coolants flowing in fully developed turbulent flow regime over or through a given geometry at a fixed velocity the ratio of Mouromtseff values (Mo) was suggested as a figure of merit [21,22]. The fluid with the highest Mo value will provide the highest heat transfer rate for cooling application. Based on the Dittus-Boelter equation, Mo value can be expressed as:

(4) $$\text{Mo}=\frac{\rho^{0.8}k^{0.67}c_{p}^{0.33}}{\mu^{0.47}};\;{\text{Mo}}_{\text{eff}}/{\text{Mo}}_{0}>1$$

Thus, the challenge in the development of nanofluids for heat transfer applications is in understanding of how micro- and macroscale interactions between the particles and the fluid affect the properties of the fluid. This requires a complex approach that accounts for changes in all important thermo-physical properties caused by introduction of nanomaterials to the fluid. It is obvious that the properties of suspensions depend on many system variables (i.e., engineering parameters) such as the nanoparticle material, concentration, size, and shape, the properties of the base fluid, and the presence of additives, surfactants, electrolyte strength, and pH. Below we discuss how each of the above parameters affects individual nanofluids properties.

### Nanofluid engineering parameters

#### Nanoparticles

Great varieties of nanoparticles are commercially available and can be used for preparation of nanofluids. Nanoparticle material, concentration, size, and shape all contribute to the nanofluid properties.

Nanoparticle material defines density, specific heat, and thermal conductivity of the solid phase contributing to nanofluids properties (subscripts p, 0, and eff refer to nanoparticle, base fluid, and nanofluid, respectively) in proportion to the volume concentration of particles (*φ*):

(5) $$\rho_{\text{eff}}=(1-\varphi )\rho_{0}+\varphi \rho_{\text{p}};$$

(6) $${\left(c_{p}\right)}_{\text{eff}}=\frac{(1-\phi ){\left(\rho c_{p}\right)}_{0}+\phi {\left(\rho c_{p}\right)}_{\text{p}}}{(1-\phi )\rho_{0}+\phi \rho_{\text{p}}};$$

(7) $$k_{\text{eff}}=k_{0}\left(\frac{k_{\text{p}}+2k_{0}+2(k_{\text{p}}-k_{0})\phi }{k_{\text{p}}+2k_{0}-(k_{\text{p}}-k_{0})\phi }\right),\;\left(\text{for the simplest case of spherical particles by EMT}\right).$$

As it was mentioned previously the materials with the higher thermal conductivity, specific heat, and density are beneficial for heat transfer.

The size of nanoparticles defines the surface-to-volume ratio and for the same volume concentrations suspension of smaller particles will have a higher area of the solid/liquid interface. Therefore, the contribution of interfacial effects will be stronger in such a suspension [23,24]. Interactions between the nanoparticles and the fluid are manifested through the interfacial thermal resistance, also known as Kapitza resistance (*R*_*k*_), which rises because interfaces act as an obstacle to heat flow and diminish the overall thermal conductivity of the system [25]. The values of Kapitza resistance are constant for the particular solid/liquid interface defined by the strength of solid-liquid interaction and can be correlated to the wetting properties of the interface [25]. When the interactions between the nanoparticle surfaces and the fluid are weak (non-wetting case) the rates of energy transfer are small resulting in relatively large values of *R*_*k*_. The overall contribution of the solid/liquid interface to the macroscopic thermal conductivity of nanofluids is negative and was found proportional to the total area of the interface, increasing with decreasing particle sizes [24,26].

The size of nanoparticles also affects the viscosity of nanofluids. In general, the viscosity increases as the volume concentration of particles increases. Studies of suspensions with the same volume concentration and material of nanoparticles but different sizes [26,27] showed that the viscosity of suspension increases as the particle size decreases. This behavior is related to formation of structured layers of the fluid along the nanoparticle interfaces that move with the particles in the flow [28]. The thicknesses of those fluid layers depend on the strength of particle-fluid interactions while the volume of immobilized fluid increases in proportion to the total area of the solid/liquid interface. The "effective volume concentration" (immobile fluid and nanoparticles) is higher in suspensions of smaller nanoparticles resulting in higher viscosity. Therefore, contributions of interfacial effects, negligible at micron particle sizes, become very important for nanoparticle suspensions. To achieve benefit for heat transfer, the suspensions of larger nanoparticles with the higher thermal conductivity and lower viscosity should be used.

A drawback of using larger nanoparticles is the potential instability of nanofluids. Rough estimation of the settling velocity of nanoparticles (*V*_s_) can be calculated from Stokes law (only accounts for gravitational and buoyant forces):

(8) $$V_{\text{s}}=\frac{2}{9}\left(\frac{\rho_{\text{p}}-\rho_{0}}{\mu }\right)r^{2}g,$$

where *g *is the gravitational acceleration. As one can see from Equation (8), the stability of a suspension (defined by lower settling rates) improves if: (a) the density of the solid material (*ρ*_p_) is close to that of the fluid (*ρ*_0_), (b) the viscosity of the suspension (*μ*) is high, and (c) the particle radius (*r*) is small.

Effects of the nanoparticles shapes on the thermal conductivity and viscosity of alumina-EG/H_2_O suspensions [24] are also strongly related to the total area of the solid/liquid interface. In nanofluids with non-spherical particles the thermal conductivity enhancements predicted by the Hamilton-Crosser equation [11] (randomly arranged elongated particles provide higher thermal conductivities than spheres, EMT [29]) are diminished by the negative contribution of the interfacial thermal resistance as the sphericity of nanoparticles decreases [24]. Elongated particles and agglomerates also result in higher viscosity at the same volume concentration as spheres due to structural limitation of rotational and transitional Brownian motion. Therefore, it can be concluded that spherical particles or low aspect ratio spheroids are more practical for achieving lower viscosities in nanofluids--the property that is highly desirable for minimizing the pumping power penalties in cooling system applications.

#### Base fluid

The influence of base fluids on the thermo-physical properties of suspensions is not very well studied and understood. However, there are few publications indicating some general trends of the base fluid effects.

Suspensions of the same Al_2_O_3 _nanoparticles in water, ethylene glycol (EG), glycerol, and pump oil showed increase in relative thermal conductivity (*k*_eff_/*k*_0_) with decrease in thermal conductivity of the base fluid [23,30]. On the other hand, the alteration of the base fluid viscosity [31] (from 4.2 to 5500 cP, by mixing two with approximately the same thermal conductivity) resulted in decrease in the thermal conductivity of the Fe_2_O_3 _suspension as the viscosity of the base fluid increased. Comparative studies of SiC suspensions in water and 50/50 ethylene glycol/water mixture with controlled particle sizes, concentration, and pH showed that relative change in thermal conductivity due to the introduction of nanoparticles is ~5% higher in EG/H_2_O than in H_2_O [27]. This effect cannot be explained simply by the lower thermal conductivity of the EG/H_2_O base fluid since the difference in enhancement values expected from EMT is less than 0.1% [15]. Therefore, the "base fluid effect" observed in different nanofluid systems is most likely related to the lower value of the interfacial thermal resistance (better wettability) in the EG/H_2_O than in the H_2_O-based nanofluids.

Relative viscosity decreases with the increase of the average particle size in both EG/H_2_O than in H_2_O-based suspensions. However, at the same volume concentration of nanoparticles relative viscosity increase is smaller in the EG/H_2_O than in H_2_O-based nanofluids, especially in suspensions of smaller nanoparticles [27]. According to the classic Einstein-Bachelor equation for hard non-interacting spheres [32], the percentage viscosity increase should be independent of the viscosity of the base fluid and only proportional to the particle volume concentration. Therefore, the experimentally observed change in viscosity increase in base fluids can be related to the difference in structure and thickness of immobilized fluid layers around the nanoparticles, affecting the effective volume concentration and ultimately the viscosity of the suspensions [24,26,27].

Since both high thermal conductivity and low viscosity increases in nanofluids are important for heat transfer performance, the nanofluids prepared from more viscous base fluids will have greater potential for practical applications.

Viscosity increase in nanofluids was shown to depend not only on the type of the base fluid, but also on the pH value (in protonic fluids) that establishes zeta potential (charge at the particle's slipping plane). Particles of the same charge repel each other minimizing the particle-particle interactions that strongly affect viscosity [24,26,33]. It was demonstrated that the viscosity of the alumina-based nanofluids can be decreased by 31% by only adjusting the pH of the suspension without affecting the thermal conductivity [24]. Nanoparticles in suspensions can be well-dispersed (particles move independently) or agglomerated (ensembles of particles move together). Depending on the particle concentration and the magnitude of particle-particle interactions that are affected by pH, surfactant additives and particle size and shape, a dispersion/agglomeration equilibrium establishes in nanoparticle suspension. Extended agglomerates can provide increased thermal conductivity as described in the literature [34,35], but agglomeration and clustering of nanoparticles result in undesirable viscosity increase and/or settling of suspensions.

Introduction of other additives (salts and surfactants) may also affect the zeta potential at the particle surfaces. Non-ionic surfactants provide steric insulation of nanoparticles preventing Van der Waals interactions, while ionic surfactants may serve as both electrostatic and steric stabilization. The thermal conductivity of surfactants is significantly lower than water and ethylene glycol. Therefore, addition of such additives, while improving viscosity, typically reduces the thermal conductivity of suspension.

It should be mentioned here that all thermo-physical properties have some temperature dependence. The thermal conductivity of fluids may increase or decrease with the temperature; however, it was shown that relative enhancement in the thermal conductivity due to addition of nanoparticles remains constant [29,36]. The viscosity of most fluids strongly depends on the temperature, typically decreasing with increasing temperature. It was noted in a couple of nanofluid systems that the relative increase in viscosity is reduced as temperature rises [26,27]. The fact of constant thermal conductivity increase and viscosity decrease with temperature makes nanofluids technology very promising for high-temperature application. The density and specific heat of nanofluids change insignificantly within the practical range of current cooling applications. Stability of nanofluids could be improved with temperature increase due to increase in kinetic energy of particles, but heating also may affect the suspension stability provided by electrostatic or/and steric methods. Further studies are needed in this area.

### Systems engineering approach to nanofluids

Systems engineering is an interdisciplinary field widely used for designing and managing complex engineering project, where the properties of a system as a whole may greatly differ from the sum of the parts' properties [37]. The decision matrix approach used in this study is a semi-quantitative technique for ranking multi-dimensional nanofluid engineering options [38]. It also offers an alternative way to look at the inner workings of a nanofluid system and allows for design choices addressing the heat transfer demands of a given industrial application.

**Figure 1:**
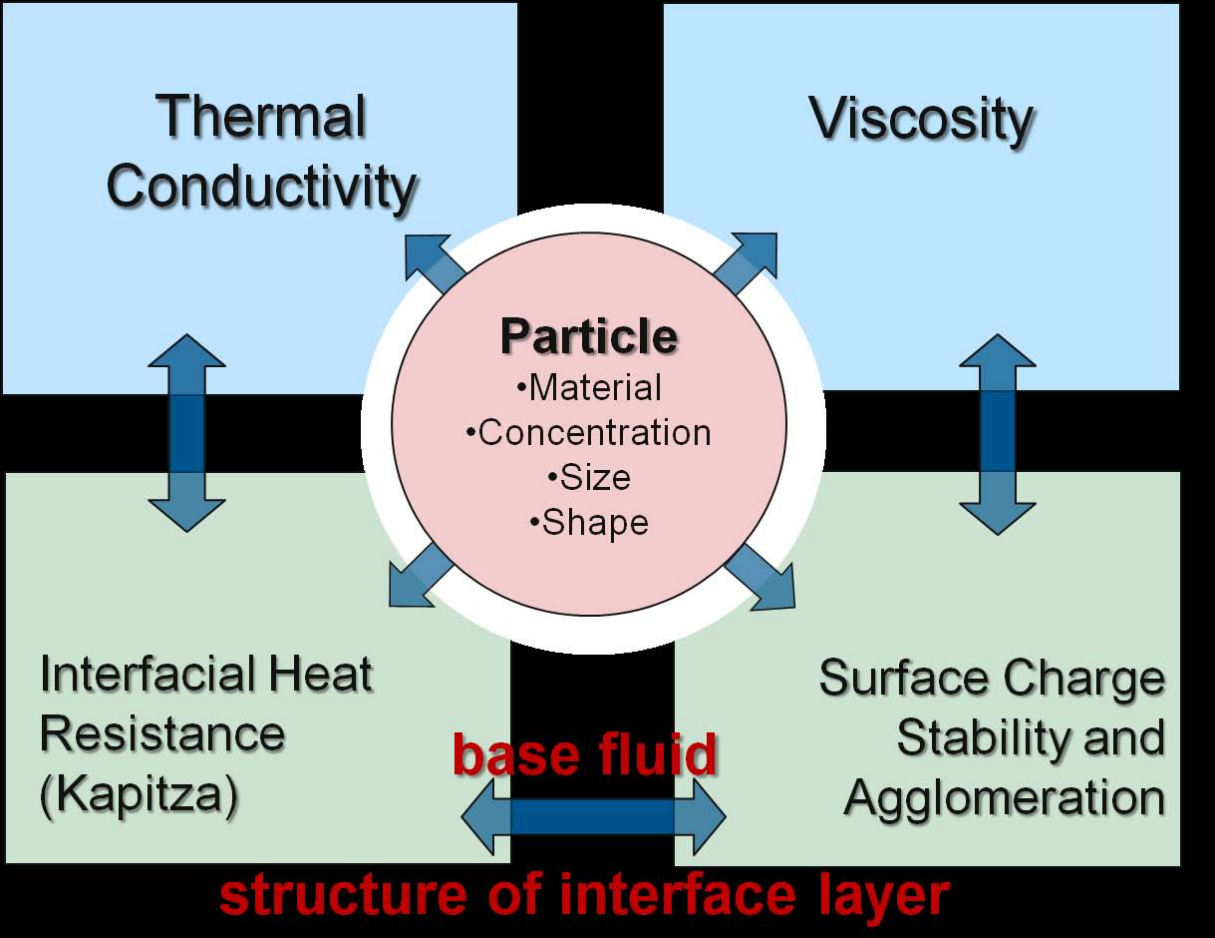
**Schematic representation of the multivariability of a nanofluid system**.

The correlations between the nanofluid engineering parameters and the nanofluid properties are schematically presented in Figure 1 as discussed in the previous sections. Due to the described complexity of the nanofluid systems, manipulation of the system performance requires identification of critical parameters and properties of nanofluids. The trends in nanoparticle suspensions observed in our experimental work and reported in the literature discussed earlier in the article are arranged in a basic decision matrix (Table 1) with each engineering parameter in a separate column and the nanofluid properties listed in rows. Each cell in the table represents the strength of the effect of a particular parameter to the nanofluid's property with "x", "▲", "○", and "■" indicating no, weak, medium, and strong dependence, respectively, that were scored as 0.0, 0.25, 0.5, and 1.0 correspondingly [38]. The relative importance of each nanofluid parameter can be estimated as a sum of the gained scores (Table 1). Based on that the nanofluid engineering parameters can be arranged by the decreasing importance for the heat transfer performance: particle concentration > base fluid > nanoparticle size > nanoparticle material ≈ surface charge > temperature ≈ particle shape > additives > Kapitza resistance. This is an approximate ranking of engineering parameters that assumes equal and independent weight of each of the nanofluid properties contributing to thermal transport. The advantage of this approach to decision making in nanofluid engineering is that subjective opinions about the importance of one nanofluid parameter versus another can be made more objective.

**Table 1:** Systems engineering approach to nanofluid design.

	ENGINEERING PARAMETERS	Nanoparticle material	Nanoparticle concentration	Nanoparticle shape	Nanoparticle size	Base fluid	Zeta potential/fluid pH	Kapitza resistance	Additives	Temperature
NANOFLUID PROPERTIES										

**Stability**	⇑	▲	▲	▲	■↓	○	■	x	■	?

**Density**	⇑	■	■↑	x	x	■	x	x	x	x

**Specific Heat**	⇑	■	■↓	x	x	■	x	x	x	▲

**Thermal Conductivity**	⇑	○	■↑	○	■↑	▲	○	■↓	▲	○

**Viscosity**	⇓	▲	■↓	■	■↓	■↑	■	x	○	■

**Heat Transfer Coefficient**	⇑	■	■↑*	■	■↑	■	■	■↓	○	■

**Pumping Power Penalty**	⇓	x	■	■	■↑	■	■	x	○	■

										

**Relative Importance**		4.0	6.25	3.75	5.0	5.25	4.0	2.0	2.75	3.75

Symbols:

■- strong dependence; ○- medium dependence; ▲- weak dependence; x - no dependence; ? - unknown or varies from system to system; ⇑ - larger the better;⇓ - smaller the better; ↑- increase with increase in parameter; ↓- decrease with increase in parameter; *-within the linear property increase.

Applications of the decision matrix (Table 1) are not limited to the design of new nanofluids; it also can be used as guidance for improving the performance of existing nanoparticle suspensions. While the particle material, size, shape, concentration, and the base fluid parameters are fixed in a given nanofluid, the cooling performance still can be improved by remaining adjustable nanofluid parameters in order of their relative importance, i.e., by adjusting the zeta potential and/or by increasing the test/operation temperatures in the above case. Further studies are needed to define the weighted importance and sensitivity of each nanofluid property contributing to the heat transfer.

## Summary

The article first identifies the thermo-physical properties of nanofluids that are important for heat transfer using the fluid dynamics-based cooling efficiency criteria for single-phase fluids. Then the nanofluid engineering parameters are reviewed in regards to their influence on the thermo-physical properties of nanoparticle suspensions. The individual nanofluid parameter-property correlations are summarized and analyzed using the system engineering approach, which allows identifying the most influential nanofluid parameters. The relative importance of engineering parameters resulted from such analysis suggests the potential nanofluid design options. Importantly, the criteria are not weighted to allow a quick selection process. The nanoparticle concentration, base fluid, and particle size appear to be the most influential parameters for improving the heat transfer efficiency of nanofluid.

## Abbreviations

EG: ethylene glycol; EMT: effective medium theory.

## Competing interests

The authors declare that they have no competing interests.

## Authors' contributions

ET carried out the preparation and testing of thermo-physical properties of nanofluids and drafted the manuscript. WY carried out the testing of the heat transfer coefficients of the fluids and developed a basis for the proper comparison of the heat transfer properties. DF participated in the design of the study and analysis of cooling efficiency. DS and JR participated in the design and coordination of the study and discussion of the results. All authors read and approved the final manuscript.

## References

[B1] Ozerinc S Kakac S Yazicioglu AG Enhanced thermal conductivity of nanofluids: a state-of-the-art review Microfluid Nanofluid 2010 8 2 145 170 10.1007/s10404-009-0524-4

[B2] Chandrasekar M Suresh S A review on the mechanisms of heat transport in nanofluids Heat Transf Eng 2009 30 14 1136 1150 10.1080/01457630902972744

[B3] Li YJ Zhou JE Tung S Schneider E Xi SQ A review on development of nanofluid preparation and characterization Powder Technol 2009 196 2 89 101 10.1016/j.powtec.2009.07.025

[B4] Yu W France DM Routbort JL Choi SUS Review and comparison of nanofluid thermal conductivity and heat transfer enhancements Heat Transf Eng 2008 29 5 432 460 10.1080/01457630701850851

[B5] Cheng LX Bandarra EP Thome JR Nanofluid two-phase flow and thermal physics: a new research frontier of nanotechnology and its challenges J Nanosci Nanotechnol 2008 8 7 3315 3332 10.1166/jnn.2008.413 19051876

[B6] Murshed SMS Leong KC Yang C Thermophysical and electrokinetic properties of nanofluids--a critical review Appl Therm Eng 2008 28 17-18 2109 2125 10.1016/j.applthermaleng.2008.01.005

[B7] Choi SUS Nanofluids: from vision to reality through research J Heat Transf Trans ASME 2009 131 3 033106 (9 pp) 10.1115/1.3056479

[B8] Wen DS Lin GP Vafaei S Zhang K Review of nanofluids for heat transfer applications Particuology 2009 7 2 141 150 10.1016/j.partic.2009.01.007

[B9] Wang XQ Mujumdar AS Heat transfer characteristics of nanofluids: a review Int J Therm Sci 2007 46 1 1 19 10.1016/j.ijthermalsci.2006.06.010

[B10] Maxwell JC A Treatise on Electricity and Magnetism 1873 Oxford, UK: Clarendon

[B11] Hamilton RL Crosser OK Thermal conductivity of heterogeneous two-component systems Ind Eng Chem Fundam 1962 1 3 187 191 10.1021/i160003a005

[B12] Buongiorno J Venerus DC Prabhat N McKrell T Townsend J Christianson R Tolmachev YV Keblinski P Hu LW Alvarado JL Bang IC Bishnoi SW Bonetti M Botz F Cecere A Chang Y Chen G Chen H Chung SJ Chyu MK Das SK Paola RD Ding Y Dubois F Dzido G Eapen J Escher W Funfschilling D Galand Q Gao J Gharagozloo PE Goodson KE Gutierrez JG Hong H Horton M Hwang KS Iorio CS Jang SP Jarzebski AB Jiang Y Jin L Kabelac S Kamath A Kedzierski MA Kieng LG Kim C Kim JH Kim S Lee SH Leong KC Manna I Michel B Ni R Patel HE Philip J Poulikakos D Reynaud C Savino R Singh PK Song P Sundararajan T Timofeeva EV Tritcak T Turanov AN Vaerenbergh SV Wen D Witharana S Yang C Yeh WH Zhao XZ Zhou SQ A benchmark study on the thermal conductivity of nanofluids J Appl Phys 2009 106 9 094312 10.1063/1.3245330

[B13] Kabelac S Kuhnke JF Heat transfer mechanisms in nanofluids--experiments and theory Annals of the Assembly for International Heat Transfer Conference 2006 13 KN 11

[B14] Trisaksri V Wongwises S Critical review of heat transfer characteristics of nanofluids Renew Sustain Energy Rev 2007 11 3 512 523 10.1016/j.rser.2005.01.010

[B15] Yu W France DM Singh D Timofeeva EV Smith DS Routbort JL Mechanisms and models of effective thermal conductivities of nanofluids J Nanosci Nanotechnol 2010 10 1 26 10.1166/jnn.2010.1484 21125818

[B16] Yu W France DM Timofeeva EV Singh D Routbort JL Thermophysical property-related comparison criteria for nanofluid heat transfer enhancement in turbulent flow Appl Phys Lett 2010 96 213109 3 10.1063/1.3435487

[B17] Etherington H (Ed) Nuclear Engineering Handbook 1958 New York, USA: McGraw-Hill Book Company, Inc

[B18] Prasher R Song D Wang JL Phelan P Measurements of nanofluid viscosity and its implications for thermal applications Appl Phys Lett 2006 89 13 133108 133110 10.1063/1.2356113

[B19] Murshed SMS Leong KC Yang C Investigations of thermal conductivity and viscosity of nanofluids Int J Therm Sci 2008 47 5 560 568 10.1016/j.ijthermalsci.2007.05.004

[B20] Routbort JL Singh D Timofeeva EV Yu W France DM Pumping power of nanofluids in a flowing system J Nanopart Res 2011 12 published online

[B21] Mouromtseff IE Water and forced-air cooling of vacuum tubes Proceedings of the Institute of Radio Engineers 1942 190 205

[B22] Simons RE Comparing heat transfer rates of liquid coolants using the Mouromtseff number Electron Cooling 2006 12 http://electronics-cooling.com/articles/2006/2006_may_cc.php

[B23] Xie HQ Wang JC Xi TG Liu Y Ai F Wu QR Thermal conductivity enhancement of suspensions containing nanosized alumina particles J Appl Phys 2002 91 7 4568 4572 10.1063/1.1454184

[B24] Timofeeva EV Routbort JL Singh D Particle shape effects on thermophysical properties of alumina nanofluids J Appl Phys 2009 106 014304 (10 pp) 10.1063/1.3155999

[B25] Barrat JL Chiaruttini F Kapitza resistance at the liquid-solid interface Mol Phys 2003 101 1605 1610 10.1080/0026897031000068578

[B26] Timofeeva EV Smith DS Yu W France DM Singh D Routbort JL The particle size and interfacial effects on thermo-physical and heat transfer characteristics of water based a-SiC nanofluids Nanotechnology 2010 21 21 215703 215713 10.1088/0957-4484/21/21/215703 20431197

[B27] Timofeeva EV Yu W France DM Singh D Routbort JL Base fluid and temperature effects on the heat transfer characteristics of SiC in EG/H_2_O and H_2_O nanofluids J Appl Phys 2011 109 014914 (5 pp) 10.1063/1.3524274

[B28] Li L Zhang YW Ma HB Yang M An investigation of molecular layering at the liquid-solid interface in nanofluids by molecular dynamics simulation Phys Lett A 2008 372 25 4541 4544 10.1016/j.physleta.2008.04.046

[B29] Timofeeva EV Gavrilov AN McCloskey JM Tolmachev YV Sprunt S Lopatina LM Selinger JV Thermal conductivity and particle agglomeration in alumina nanofluids: experiment and theory Phys Rev E 2007 76 061203 061216 10.1103/PhysRevE.76.061203 18233838

[B30] Xie HQ Wang JC Xi TG Liu Y Ai F Dependence of the thermal conductivity of nanoparticle-fluid mixture on the base fluid J Mater Sci Lett 2002 21 19 1469 1471 10.1023/A:1020060324472

[B31] Tsai TH Kuo LS Chen PH Yang CT Effect of viscosity of base fluid on thermal conductivity of nanofluids Appl Phys Lett 2008 93 23 233121 (3 pp) 10.1063/1.3046732

[B32] Vold RD Vold MJ Colloid and Interface Chemistry 1983 Reading, MA: Addison-Wesley Publishing Company, Inc

[B33] Zhao JF Luo ZY Ni MJ Cen KF Dependence of nanofluid viscosity on particle size and pH value Chin Phys Lett 2009 26 6 066202 (3 pp) 10.1088/0256-307X/26/6/066202

[B34] Prasher R Evans W Meakin P Fish J Phelan P Keblinski P Effect of aggregation on thermal conduction in colloidal nanofluids Appl Phys Lett 2006 89 14 143119 (3 pp) 10.1063/1.2360229

[B35] Eapen J Rusconi R Piazza R Yip S The classical nature of thermal conduction in nanofluids J Heat Transf Trans ASME 2010 132 10 102402 (14 pp) 10.1115/1.4001304

[B36] Singh D Timofeeva E Yu W Routbort J France D Smith D Lopez-Cepero JM An investigation of silicon carbide-water nanofluid for heat transfer applications J Appl Phys 2009 105 6 064306 (6 pp) 10.1063/1.3082094

[B37] Kossiakoff A Sweet WN Sage A Systems engineering: principles and practice Systems Engineering and Management 2003 New York: Wiley-IEEE 463

[B38] Pugh S Clausing D Andrade R Creating Innovative Products Using Total Design: The Living Legacy of Stuart Pugh 1996 Reading, MA: Addison-Wesley Pub. Co

